# Interproximal contact areas of primary molars based on OXIS classification – a two centre cross sectional study

**DOI:** 10.12688/wellcomeopenres.16424.2

**Published:** 2021-02-03

**Authors:** Tarun Walia, M Kirthiga, Carel Brigi, M S Muthu, Ruba Odeh, Vijay Pakash Mathur, Steven Rodrigues

**Affiliations:** 1 Department of Clinical Sciences, Ajman University, Ajman, Ajman, 346, United Arab Emirates; 2 Centre for Early Childhood Caries Research, Department of Pediatric and Preventive Dentistry, Faculty of Dental Sciences, Sri Ramachandra Institute of Higher Education and Research, Chennai, Tamilnadu, 600116, India; 3 College of Dentistry, Ajman University, Ajman, 346, United Arab Emirates; 4 Division of Pedodontics and Preventive Dentistry Centre for Dental Education and Research,, All India Institute of Medical Sciences, Delhi, 110029, India; 5 Division of Pedodontics and Preventive Dentistry,, Goa Dental College, Goa, 403202, India

**Keywords:** CHILD, OXIS CONTACTS, DECIDUOUS MOLARS, CROSS SECTIONAL

## Abstract

**Background: **Contact areas in primary teeth are known to be broader, flatter and situated farther gingivally than permanent teeth. ****The purpose of this study was to assess the prevalence of OXIS contact areas between primary molars using die models of children from two different ethnic populations. The research question of the present study is “What is the prevalence OXIS contact areas of primary molars in the populations studied?”.

**Methods: **A cross-sectional study was planned in a representative sample of 200 caries-free contact areas among children aged 3-6 years in two centers, Ajman and Puducherry. Data collection was performed from sectional or full-arch die stone models. The contacts were assessed according to OXIS classification by a single calibrated examiner at Center 2. Prevalence was expressed as numbers and percentages. The Chi-square test was applied to determine the association of OXIS contacts across genders and arches.

**Results:** The prevalence of O, X, I, and S contacts were 19%, 22.5%, 53%, and 5.5% in Center 1 and 6%, 1.5%, 75.5%, and 17% in Center 2, respectively. Significant results (p=0.005) were obtained in Center 1 when inter-arch comparison was performed and between the centers with respect to type of contact. No significant difference was obtained with respect to gender and OXIS contact areas.

**Conclusions:** The present study confirmed OXIS contacts in both the centers. The ‘O’ and ‘X’ types of contacts were observed more in Center 1, whereas ‘I’ and ‘S’ contacts were observed more in Center 2.

## Introduction

The anatomic and morphologic configurations of a tooth, specifically a broad, flat proximal contact area, are vital for maintenance of the stability and integrity of the dental arches and health of the supporting structures ^1– 3^. A well-contoured, firmly established proximal contact defines the gingival embrasure and the height of the interdental papilla. The types of contacts observed between primary molars are broad, flat, and situated gingivally when compared with those of permanent molars ^1, 2, 4^. The broad proximal contact areas observed in primary molars are likely to increase caries susceptibility, since self-cleansing action would be reduced due to the limited movement, leading to greater plaque accumulation ^1, 2^. This was affirmed in a Danish study conducted in 2005 in four- to six-year-old children. The study concluded that plaque accumulation, bleeding on probing, and broad contact areas between the primary molars were useful predictors for approximal caries in the primary dentition ^5^.

There is little in the literature with respect to specific types of contacts. Most studies evaluated the association of two types of contacts (open and closed) with proximal caries. Studies by Allison and Schwartz in 2003 ^6^ and Subramaniam *et al.* in 2012 ^4^ concluded that the risk for proximal caries in the posterior primary dentition is increased if contact points are closed rather than open. Regarding the shapes of contacts, Carlsen ^7^ stated that the approximal surfaces of primary molar teeth can be convex or concave in the bucco-lingual direction as well as in the occluso-cervical direction. A study by Cortes *et al.* in 2018 ^8^ concluded that the concave morphology of approximal surfaces of primary molars can predict future caries lesions. In this study, the morphology of the interproximal surfaces between the distal surfaces of the first molar and the mesial surfaces of the second molar was scored as four variants: concave-concave, concave-convex, convex-concave, and convex-convex. However, the specific types of contact areas between primary molars were first established in 2018 as O (open contact), X (point contact), I (straight contact), and S (curved contact) types, and the OXIS classification was proposed ^9^. This was later confirmed in a population-based study of 1,119 schoolchildren aged 3–4 years ^10^.

 It is indisputable that the role of OXIS contacts is significant to an understanding of the mechanism of proximal caries. One of the main limitations of the clinical study conducted in Puducherry was that the OXIS contacts could not be generalized to other ethnic populations and needed to be confirmed in future studies ^10^. Although the presence of OXIS was established in previous studies, there were statistically significant differences in the prevalence of individual contact types among those studies ^9– 11^. Therefore, the aim of the present study was to investigate the prevalence of OXIS contact areas of primary molars in study models of children aged between three and six years in two different ethnic populations.

## Methods

### Ethics approval and consent

The study protocol was approved by the Research Ethics Committee of Ajman University (Reference number, P-F-H-19-01-14) and the Institutional Ethics Committee of Sri Ramachandra Institute of Higher Education & Research (IEC-NI/16/AUG/55/54) In addition, informed written parental consent was obtained from those children involved in the study.

### Study population

Center 1 and Center 2 were Ajman and Puducherry, respectively.

Ajman is the capital of the emirate of Ajman in the United Arab Emirates, located along the Persian Gulf. According to the 2017 census, Ajman had an estimated total population of 113,242, among which 19,024 were aged 14 years or less. The ethnic composition of Ajman is mixed, i.e., 41.5% South Asian (28.5% Indian and 13% Pakistani), 17.5% Emirati (local population), 21% Palestinian, 14% Jordanian, and 6% Egyptian.

Puducherry is one of the seven Union Territories of India. According to the 2011 census, the city has an estimated population of 1,247,953, including 132,858 children (aged 0–6 years). It is divided into five zones, with 113 private schools and 161 government schools that included children in the age range between three and four years according to the Directorate of School Education, Government of Puducherry. The ethnic composition of Puducherry is 100% South Asian.

### Study design and sample

A cross-sectional study was carried out with a representative sample of children aged 3–6 years who belonged to two different centers The date ranges for both centers is as given below:

Centre 1
Data recruitment & Data collection- June 2019 and January 2020
Analysis for both centers – April 2020
Centre 2
Data recruitment & Data collection- October 2019 and February 2020
Analysis for both centers – April 2020

The calculation of the sample size was based on a previously conducted study ^10^ for estimation of the prevalence of open contacts of primary molars. Thus, the sample size was calculated assuming an expected prevalence of 30% and a *z*-value of 1.96. A minimum sample size of 200 contacts from each center was determined regardless of the number of children included.

### Sampling method for Centre 1

The representative sample from Centre 1 were selected from the Out Patient Department of Ajman University by means of convenience sampling.

### Sampling method for Centre 2

From the 4,476 models of 1,119 children procured from an already existing study ^8^, 200 good-quality models from 50 children were selected by systematic random sampling for the present investigation.

### Eligibility criteria

Inclusion criteria:

- Children aged between 3 and 6 years.
- Models of children with at least one quadrant involving caries-free (International Caries Detection and Assessment System = 0) primary molars.
- Children who co-operated in the generation of impressions.
- Good-quality models (absence of porosities).
- Children whose parents provided written consent.
- Children with no visible dental plaque on the quadrant to be included for impression-making.

Exclusion criteria:

- Children with special healthcare needs.
- Children who showed the presence of developmental anomalies in the shapes and sizes of their teeth.
- Children with a severe gag reflex.

### Calibration of the examiner

Prior to the start of the study, a single pediatric dentist (KM) was extensively trained and calibrated under the supervision of an expert (MSM) to clinically evaluate the contact areas over a period of two months. The detailed process of calibration has been explained in a previously published pilot study ^10^.

### Data collection

***Center 1*.** Data were obtained by means of sectional or full-arch impressions. First, each child was clinically examined. Cotton rolls were used to clean any food debris present, after which the teeth were dried. Following this, the caries status of each tooth was recorded. The selected children were examined for dental caries according to the International Caries Detection and Assessment System (ICDAS II) criteria ^12^ with the help of a mouth mirror and CPITN probe if necessary. Teeth were initially assessed wet and then air-dried by means of a three-way syringe. The examiner assessed all the surfaces of each tooth and recorded the findings on a form. When necessary, a CPITN probe was used for the assessment of enamel breakdown. The total examination time for each child ranged from two to three minutes. Based on the inclusion criteria, impressions for each child were made with silicone-rubber-based impression material (Zhermack SpA, Badia Polesine, Italy) and sectional or full-arch impression trays. The impressions were poured with Type IV die stone (Dentify GmbH, Engen, Germany). Of the 85 children who were selected according to the inclusion criteria, seven parents did not provide written consent, which led to 78 children participating in the main study. Therefore, 118 die models, of which 28 were sectional (28 contacts) and 90 (180 contacts) were full-arch, were sent to the main center (Sri Ramachandra Institute of Higher Education and Research, SRIHER) in batches of four for outcome assessment.

***Center 2*.** Data collection from the models in the main center was performed from existing data in a previous study conducted among children aged 3 to 4 years in Puducherry ^8^. The clinical examination was conducted in a suitable classroom by means of a mouth mirror and probe under natural light (Type III examination). Sectional die impressions (Zetaplus C Silicone Impression material – Zhermack, Thane) were made among 1,119 caries-free children with the use of disposable trays in a school setting and poured with Type IV die stone (Kalabhai Ultrarock Die Stone, Mumbai, India).

### Assessment of the outcome

First, the quality of the models was assessed based on the inclusion criteria. From Center 1, we obtained a total of 118 die models with 206 contacts. Of the total models obtained, three models with six contacts were excluded because their casts had broken teeth or large voids. Thus, 115 models with 200 contacts were analyzed in the present study. From the second center, all 200 of the study models selected were used for outcome assessment.

The assessment of the type of contact area between the distal surface of the first primary molar and the mesial surface of the second primary molar in the selected quadrants was performed by the calibrated examiner (KM) at SRIHER and scored according to the OXIS criteria ^7^. The contact observed was scored in the form of O (open contact), X (point contact), I (straight contact), and S (curved contact), or others (if there was a different shape) as seen from an occlusal view with a minimum distance of 12 inches by means of illuminated mini dental loupes with LED light, 2.5x magnification (Keeler Ltd., Windsor, UK). The data were recorded on a custom-made sheet.

Statistical analysis was performed with the Statistical Package for Social Sciences 19 (SPSS, Chicago, IL, USA). Descriptive statistics were acquired for all variables. The prevalence of the types of contact areas was expressed in the form of numbers and percentages. Chi-square test was applied to determine the association of OXIS contacts across genders and arches within Centers 1 and 2. Chi-square test was also applied to understand the association of OXIS contacts between Centers 1 and 2.

## Results

In total, 200 contacts were included from each Center in the present study. In Center 1, 200 contacts were obtained from 78 children with a mean age of 4.45 years. In Center 2, 200 contacts were obtained from 50 children with a mean age of 3.5 years. Table 1 shows the ages and quadrant distributions of OXIS contacts in Centers 1 and 2. Almost equal numbers of quadrants were selected between the age groups of 3–4 years and 4.1–5 years.

**Table 1.** Age and quadrant distribution of OXIS contacts in Center 1 and 2.

Centre 1							Centre 2						
Age	Number of children	Number of quadrants	O (%)	X (%)	I (%)	S (%)	Age	Number of children	Number of quadrants	O (%)	X (%)	I (%)	S (%)
3–4 years	37	92	14 (15.2)	21 (22.8)	53 (57.8)	4 (4.3)	3–4 years	50	200	12 (6)	3 (1.5)	151 (75.5)	3 (17)
4.1–5 years	31	95	19 (20)	20 (21.1)	50 (52.7)	6 (6.3)	4.1–5 years						
5.1–6 years	10	13	5 (38.5)	4 (30.8)	3 (23.1)	1(7.7)	5.1–6 years						
Total	78	200	38	45	106	11	Total	50	200	12	3	151	3

O, open contact; X, point contact; I, straight contact; S, curved contact.

### Prevalence and percentages

Table 2 summarizes the prevalence and percentages of contacts between the primary molars according to arch and side.

**Table 2.** Prevalence and percentages of primary molar contacts by arch and side.

	Center 1 (Ajman)					Center 2 (Puducherry)				
	Maxilla		Mandible		Total (%)	Maxilla		Mandible		Total (%)
	Right (%)	Left (%)	Right (%)	Left (%)		Right (%)	Left (%)	Right (%)	Left (%)	
O	13 (6.5%)	10 (5%)	8 (4%)	7 (3.5%)	**38** **(19%)**	4 (2%)	3 (1.5%)	3 (1.5%)	2 (1%)	**12** **(6%)**
X	11 (5.5%)	15 (7.5%)	10 (5%)	9 (4.5%)	**45** **(22.5%)**	1 (0.5%)	0	0	2 (1%)	**3** **(1.5%)**
I	20 (10%)	15 (7.5%)	35 (17.5%)	36 (18%)	**106** **(53%)**	37 (18.5%)	37 (18.5%)	39 (19.5)	38 (19.0%)	**151** **(75.5%)**
S	4 (2%)	1 (0.5%)	1 (0.5%)	5 (2.5%)	**11** **(5.5%)**	8 (4%)	10 (5%)	8 (4%)	8 (4%)	**34** **(17%)**
	48	41	54	57	**200**	50	50	50	50	**200**

O, open contact; X, point contact; I, straight contact; S, curved contact.

### Frequency of contacts

***Center 1*.** The most common contact observed in both the maxilla and the mandible was ‘I’, followed by ‘X’, ‘O’, and ‘S’. Overall, the most common contact was ‘I’ (53%), followed by ‘X’ (22.5%), then ‘O’ (19%), and finally ‘S’ (5.5%).

**Figure 1.**
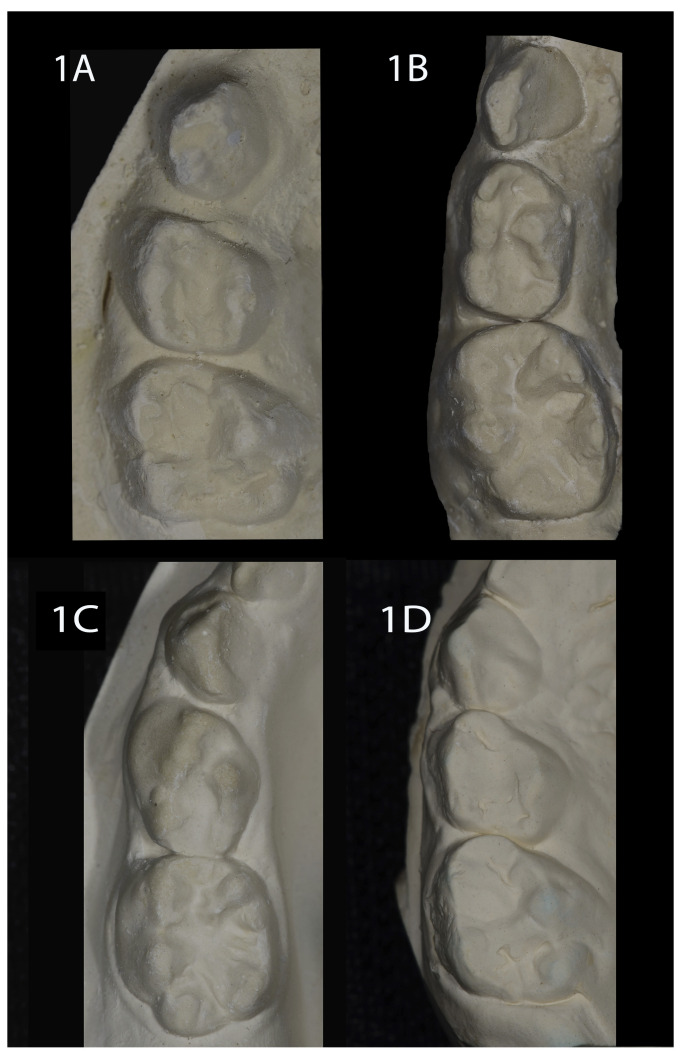
Sectional impressions of contact areas of primary molars from Centre 1 and Centre 2. **A**) Depicting “O” or open type of contact in the maxilla from Centre 2; **B**) depicting “X” or point type of contact in the mandible from Centre 2; **C**) depicting “I” or straight type of contact in the mandible from Centre 1; and **D**) depicting “S” or curved type of contact in the maxilla from Centre 1.

***Center 2*.** The most common contact observed in both the maxilla and the mandible was ‘I’, followed by ‘S’, ‘O’, and ‘X’. Overall, the most common contact was ‘I’ (75.5%), followed by ‘S’ (17%), then ‘O’ (6%), and finally ‘X’ (1.5%). Figure 1 shows sectional impressions of contact areas of primary molars from Center 1 and Center 2.

### Inter-arch, gender, and inter-center comparisons

When the inter-arch comparison was performed for OXIS types of contacts, a statistically significant difference was observed in Center 1 ( *P*=0.005). However, this comparison was not found to be statistically significant in Center 2 ( *P*=0.839). When OXIS contacts were compared between genders, no statistically significant results were obtained with respect to both centers.

Statistically significant results ( *P*<0.005) were observed with respect to frequency of types of contacts between Center 1 and Center 2.

## Discussion

The present cross-sectional two-center study is the first of its kind where variations in the prevalence of OXIS contacts were studied in two different countries. Of the two studies ^10, 11^ performed recently using the OXIS classification, only one ^10^ was an epidemiological study, performed in the population of Puducherry. Therefore, OXIS contacts could not be generalized to all the ethnic populations, and this laid the scientific base for the present investigation, which confirmed OXIS contacts in both the chosen centers. Further, the study also confirmed that no other shape or type of contact area existed other than OXIS, although there was a provision called ‘others’ if any other shape was observed. This finding was in agreement with those of the previous epidemiological study ^10^.

### Sample size and ages

In this study, equal numbers of models were included from both centers. To match the sample from Center 1, 200 samples were chosen from the 4,476 available models from a previous study ^10^ in Center 2. The sample size evaluated in the previous studies included 74 contacts ^9^, 1,343 contacts ^11^, and 4,476 contacts ^10^ in the epidemiological study. The age distribution chosen in the present study was 3–6 years in the first Center, with the majority of children (87%) aged between three and five years. In Center 2, the children were 3–4 years old. Nevertheless, all the children selected had exclusively primary dentition.

### Method of scoring OXIS contacts

The present study used die models (sectional/full-arch) for scoring OXIS contacts. Center 1 used sectional and full-arch die models, while Center 2 utilized only the sectional models. In the previous epidemiological study ^10^ performed in 1,119 children aged 3–4 years, OXIS contacts were assessed by clinical examination. Models were also made, but only for record-keeping purposes. In addition, the closed or open nature of the contact point was first assessed by the passing of dental floss through the interproximal contact point ^6^, after which the OXIS criteria were applied. This was not possible in the present study, since only die models were used for the outcome assessment, and not clinical examination. A previous study ^8^ used stereomicroscopic (1.6x magnification) images of the models taken from an occlusal-cervical direction at a right angle. The present study utilized dental loupes to assess the contact area types, which provided only two-dimensional views. Another recent study ^11^ was conducted to correlate between the types of OXIS contact areas by cone beam computed tomography (CBCT) and those by clinical photographs. The study revealed a correlation of 0.958, indicating that a two-dimensional evaluation was sufficient to score the types of OXIS contact areas. Therefore, in the current study, only visual examination of the die models was used for diagnosis of the types of contact areas between the primary molars.

### Frequency of OXIS contacts

The percentage of ‘O’-type contacts was 19% in Center 1 in contrast to 6% in Center 2. The result observed in Center 2 was similar to that reported in a previous study ^10^, but differed from other studies ^9, 11^. The present study also showed more type ‘O’ contacts in the maxilla than in the mandible in both centers. This finding was in agreement with other studies conducted earlier ^4, 13^. According to Baume, spaces between first and second primary molars disappear between 2.5 and 3.5 years of age, especially in the mandible ^14^.

The percentage of ‘X’ contacts observed was 22.5% in Center 1 in contrast to 1.5% in Center 2. The results found in Center 2 were similar to those from a previous study ^9^ but differed from those of other studies ^10, 11^.

The percentage of ‘I’ contacts observed was 53.0% in Center 1 in contrast to 75.5% in Center 2. The result observed in Center 1 was comparable with the results observed in all the previous studies ^9– 11^.

The percentage of ‘S’ contacts observed was 5.5% in Center 1 in contrast to 17.0% in Center 2. This result differed from those found in the previous studies ^9– 11^.

### Overall frequency of contacts

Overall, the order from the most to the least common types of contact areas in Center 1 and Center 2 was I>X>O>S and I>S>O>X, respectively. The most common contact observed in both centers was type ‘I’. This finding was in agreement with those of the previous studies by the same group of authors ^9– 11^. The least common contacts observed in the present study were ‘S’ and ‘X’ in Center 1 and Center 2, respectively. In addition, the outcome for the least common contact was different in all the studies conducted previously. This difference could be attributed to the variations in the ethnicity of the populations, sample size, and the ages chosen. The percentages of closed contacts (X, I, S) in the present study were 81% and 88% in Centers 1 and 2, respectively. This finding was similar to those of previous studies, where prevalences of 94.1% ^10^, 90.5% ^9^, 90% ^4^, and 84% ^6^ were reported.

### Inter-arch and gender comparisons

Statistically significant results ( *P*=0.005) were obtained when the maxilla was compared with the mandible (inter-arch comparison) in Center 1. However, no statistically significant results were observed with respect to gender. Both these findings were correlated with respect to the previous studies performed in this area ^10, 11^.

### Difference in frequency between centers

A remarkable finding of the present study with respect to the frequency of contacts was that the percentages of ‘O’ and ‘X’ contacts were 19% and 22.5% in Center 1, in contrast to 6% and 1.5% in Center 2. Further, the percentages of ‘I’ and ‘S’ contacts were 53% and 5.5% in Center 1, in contrast to 75.5% and 17% in Center 2. This statistically significant difference ( *P*<0.005) could be explained by two major reasons: first, by the variation in ethnic populations; and second, by the difference in the age groups included in both centers. The ethnic population in Center 1 was mixed, with only 17.5% of the local (Emirati) population. In Center 1, the percentages of ‘O’ and ‘X’ contacts were greater in the 5.1- to 6-year-old age group than in the 3- to 4- and 4.1- to 5-year-old groups, where ‘I’ was the most common contact type. In the older age group (5–6 years), an increase in jaw dimensions to accommodate developing permanent first molars could be expected. Hence, the number of ‘O’ and ‘X’ contacts could have been increased in this center.

### Clinical implications and future research

Another interesting hypothesis from the present study is the change in the types of contacts with age and its relationship with the increase in jaw size in different ethnic populations. Although the contact area between the first and second primary molars is usually established around the third and fourth years of life ^8^, there is no evidence available regarding the change in contacts over a period of time in the populations studied. The type of contact is transitory and may change due to eruption and growth of the jaws of the child ^6^. The same child could have different contacts at different ages starting from 2.5 years until the age of six.

The most significant clinical implication of the type of contact area is that it could be a risk factor for the occurrence of proximal caries between the primary molars and hence should be included in the caries risk assessment for children ^10^. In our study, it is rational to hypothesize that the ‘I’ and ‘S’ types of contacts would be inaccessible for mechanical cleansing when compared with the ‘O’ and ‘X’ types ^10^. This could lead to greater plaque accumulation and retention below the contact area, further leading to proximal caries. The present study reports ‘I’ as the most common contact in both centers. However, the ‘S’ type prevalence was 5.5% in Center 1 and 17% in Center 2.

Future studies should use a standardized methodology to address the prevalence of OXIS contacts in different ethnic populations. The dynamic changes in the establishment of a contact area with changes in arch length as children age should also be studied in different ethnic populations. Future investigations by cohort studies are required to understand the risks associated with each type of contact for the development of early childhood caries.

One limitation of the study was that the data from Center 1 were acquired in a mixed ethnic population. Hence, extrapolation of these results to the general population should be done with caution.

The present study confirmed OXIS contacts in both centers, although with considerable differences in frequency. The ‘O’ and ‘X’ types of contacts were observed more in Center 1 than in Center 2, whereas ‘I’ and ‘S’ contacts were observed more in Center 2.

## Data availability

Open Science Framework: OXIS contact areas of primary molars – a two center cross-sectional study, https://doi.org/10.17605/OSF.IO/ZBGMK ^15^

Data are available under the terms of the Creative Commons Zero "No rights reserved" data waiver (CC0 1.0 Public domain dedication).
