# Supplementary material for: Interproximal contact areas of primary molars based on OXIS classification – a two centre cross sectional study
Source: Wellcome Open Res. 2021 Feb 3;5:285. Originally published 2020 Dec 7. [Version 2] doi: 10.12688/wellcomeopenres.16424.2 (PMC7836030; doi:10.12688/wellcomeopenres.16424.2)
Supplement: Peer review history

# Peer review: Reviewer response for version 1

**DOI:** 10.21956/wellcomeopenres.18071.r41873

**Published:** 2021-01-22

**Authors:** Sapna Hegde

**Affiliations:** 1 Sultan Al Olama Medical Center, Dubai, United Arab Emirates

- This article presents the prevalence of the types of interproximal contacts between primary molar teeth as described in the OXIS classification.
- I would suggest a revision of the title to something like 'Interproximal contact areas based on OXIS classification...'.
- Please provide suitable explanation for comparing 2 centers when the same is not between comparable samples (as you have stated, one comprises of a pure ethnicity and other, mixed ethnicities).

Is the work clearly and accurately presented and does it cite the current literature?

Yes

If applicable, is the statistical analysis and its interpretation appropriate?

Yes

Are all the source data underlying the results available to ensure full reproducibility?

Yes

Is the study design appropriate and is the work technically sound?

Yes

Are the conclusions drawn adequately supported by the results?

Yes

Are sufficient details of methods and analysis provided to allow replication by others?

Yes

Reviewer Expertise:

Pediatric dentistry, Special care dentistry

I confirm that I have read this submission and believe that I have an appropriate level of expertise to confirm that it is of an acceptable scientific standard.

# Response

**Published:** 2021-01-28

**Authors:** M KIRTHIGA

**Affiliations:** Dr., India

- Based on the reviewer suggestion we plan to reframe the title from “OXIS contact areas of primary molars – a two centre cross-sectional study" to “Interproximal contact areas of primary molars based on OXIS classification – a two centre cross sectional study.”
- We chose the centres based on our convenience. Further, the contact areas of primary molar based on OXIS classification had not been performed in the population of Ajman. Hence we chose this centre. This has also been mentioned as the limitation of our study.

# Peer review: Reviewer response for version 1

**DOI:** 10.21956/wellcomeopenres.18071.r41872

**Published:** 2021-01-08

**Authors:** Lourdes Aparecida Martins dos Santos-Pinto

**Affiliations:** 1 School of Dentistry, Department of Pediatric Dentistry, Universidade Estadual Paulista – Unesp, Araraquara, Brazil

- I suggest not using the acronym OXIS in the title. It could be: Types of interproximal contacts of primary molars…. OXIS is a classification and may not be familiar to the readers.
- The abstract accurately summarizes the contents of the manuscript and the introduction provides enough information if the readers are not familiar with the topic.
- Some details about calibration will be interesting, especially kappa’s value.

Is the work clearly and accurately presented and does it cite the current literature?

Yes

If applicable, is the statistical analysis and its interpretation appropriate?

Yes

Are all the source data underlying the results available to ensure full reproducibility?

Yes

Is the study design appropriate and is the work technically sound?

Yes

Are the conclusions drawn adequately supported by the results?

Yes

Are sufficient details of methods and analysis provided to allow replication by others?

Yes

Reviewer Expertise:

Pediatric dentistry

I confirm that I have read this submission and believe that I have an appropriate level of expertise to confirm that it is of an acceptable scientific standard.

# Response

**Published:** 2021-01-28

**Authors:** M KIRTHIGA

**Affiliations:** Dr., India

- Since a similar suggested in the title was provided by another reviewer we plan to reframe the title from “OXIS contact areas of primary molars – a two centre cross-sectional study" to “Interproximal contact areas of primary molars based on OXIS classification – a two centre cross sectional study.”
- The detailed process of calibration has been explained in a previously published pilot study (Kirthiga M, Muthu MS, Kayalvizhi G, *et al.*: Proposed classification for interproximal contacts of primary molars using CBCT: a pilot study [version 2; peer review: 2 approved]. *Wellcome Open Res.* 2018; **3**: 98.) Since this would be a repetition it was not mentioned again.
